# Factors associated with Myanmar women’s attitudes toward negotiating safer sexual relations

**DOI:** 10.3389/fpubh.2025.1586603

**Published:** 2025-11-20

**Authors:** Myo Tint, Napaphat Poprom, Aksara Thongprachum

**Affiliations:** Faculty of Public Health, Chiang Mai University, Chiang Mai, Thailand

**Keywords:** Myanmar women, negotiating, safer sexual relations, Myanmar demographic and health survey, factor

## Abstract

**Introduction:**

Globally, HIV and STIs remain major public health challenges, with women disproportionately affected. Myanmar is among the 35 countries contributing to 90% of new HIV infections worldwide. Addressing these challenges requires strengthening women’s ability and fostering positive attitudes toward negotiating safer sexual relations (SSR), as these factors directly influence their behaviors and capacity to protect their sexual health. This study aimed to identify factors associated with Myanmar women’s attitudes toward negotiating SSR, and examine changes in associations when additional variables were introduced.

**Method:**

This study analyzed a weighted sample of 6,127 married women aged 15 to 49 from the most recent nationally representative 2015–16 Myanmar Demographic and Health Survey (2015–16 MDHS). Descriptive analysis and both simple and multiple logistic regression analyses were applied in this study.

**Result:**

Overall, 86.5% of the sample reported positive attitudes toward negotiating SSR. Higher odds of positive attitudes were consistently observed among women who were undecided about fertility preference (aOR 1.893, 95% CI: 1.028–3.488), in the poorer (aOR 1.381, 95% CI: 1.092–1.747) and richest (aOR 1.537, 95% CI: 1.117–2.114) wealth quintiles, residing in regions (aOR 1.442, 95% CI: 1.208–1.720), living in monogamous households (aOR 1.524, 95% CI: 1.132–2.051), knowledgeable about HIV transmission (aOR 1.495, 95% CI: 1.141–1.959), and self-efficacy in refusing sex and requesting condom usage (aOR 1.388, 95% CI: 1.128–1.708, aOR 2.713, 95% CI: 2.228–3.303, respectively), even after adjusting for all variables. Interestingly, employed women were consistently less likely to report positive attitudes, even in Model 2 (aOR 0.849, 95% CI: 0.722–0.999).

**Conclusion:**

This study found that most married women in Myanmar had positive attitudes toward negotiating SSR. Additionally, the findings revealed the complexity of factors associated with their attitudes, underscoring the importance of addressing both structural and interpersonal barriers. Gender-sensitive and tailored public health interventions, including sexual and reproductive health education, are needed to help reduce HIV/STI transmission and improve reproductive health outcomes.

## 1 Introduction

The concept of safer sexual relations generally includes, but is not confined to, engaging in sexual intercourse with just one committed partner, ensuring that neither person is affected by sexually transmitted infections (STIs), and using condoms consistently (1). Negotiation for safer sex involves the discussions and agreements between partners in a sexual relationship to engage in intercourse in a way that safeguards both individuals from negative sexual health consequences (2). In sexual relationships, women are frequently expected to take on the responsibility of prioritizing safe sexual practices by initiating and leading the negotiation process with their sexual partners (3). However, men usually tend to have greater control over sexual decision-making, which often limits women’s ability to negotiate. This power imbalance can lead to negative sexual and reproductive health results, such as an increased risk of Human Immunodeficiency Virus (HIV)/STIs (2).

In 2023, roughly 39.9 million people globally were living with HIV, and girls and women constitute over half of this population (around 20.5 million) (4). Among other countries, Myanmar is part of 35 countries responsible for 90% of new HIV infections worldwide (5). Myanmar has approximately 0.28 million people living with HIV, 39.2 percent of those figures are made up of females aged 15 and above in 2023 (6). Young women are twice as likely to be infected compared to men at their age, especially those involved in sex work or who use drugs (7). Additionally, there are more than 1 million people worldwide who contract sexually transmitted infections (STIs) daily, and an estimated 374 million people each year (8), and it was also mentioned that the prevalence of Syphilis and other STIs is still high in Myanmar (9).

To reduce such sexual and reproductive public health problems and promote safer sexual practice, the role of negotiation for SSR is critical (2, 10), especially in patriarchal environments where gender norms and power imbalances hinder women’s ability to make decisions regarding their sexual and reproductive well-being (2, 10, 11). Some studies revealed that possessing positive attitudes about negotiation for safer sex is crucial for women to engage and communicate effectively with their partners about sexual risks (12, 13) as it can minimize the risk of public health-related burdens like STIs, including HIV, and unwanted pregnancies (10, 13–15). The Theory of Planned Behavior also mentions that attitudes play a critical role in shaping humans’ intentions toward a specific behavior (16, 17). In many low- and middle-income countries, women’s inability to negotiate for safer sex significantly increases their risk of contracting STIs (15, 18). In addition, this risk is also associated with sexual attitudes, beliefs, and gender-power dynamics in heterosexual relationships (19).

Myanmar, as a patriarchal society (20), still maintains cultural norms that foster a strong sense of reserve in discussing sexual matters, even within marriage (21). Myanmar women often face challenges when it comes to negotiating the use of condoms with their partners to protect themselves from HIV, even if they suspect their partners of being involved in extramarital affairs (22). Additionally, it is also important to recognize that in patriarchal environments, a woman’s awareness of STIs and their consequences may not lead to effective negotiation for safer sex. This is often due to significant social and structural barriers, such as sexual restraint influenced by religious values, deeply rooted gender norms, unequal power dynamics, and the threat of domestic violence, all of which can impede her ability to assert her sexual health rights (15) spotlighting the importance of recognizing and addressing all factors shaping women’s attitudes and influencing their ability to negotiate for SSR.

Other previous studies found that women’s attitudes toward negotiating SSR are a complex mechanism and are affected by varied factors, even though the findings are different depending on different contexts (10, 13, 15, 23). While comparable studies on this topic were done in other nations, there is a limited study in Myanmar. Overlooking and failing to tackle these influencing factors will slow down progress toward achieving the Sustainable Development Goal (SDG) of ending the Acquired Immunodeficiency Syndrome (AIDS) epidemic by 2030 (24).

This study will be the first one to fill this knowledge gap in research and the public health domain using the nationally represented dataset from the 2015–16 MDHS. By examining these factors, the study ultimately intends to inform evidence-based interventions among policymakers and healthcare professionals to consider all determinants that affect women’s sexual and reproductive health and formulate integrated strategies to promote public health outcomes and reduce gender disparities in Myanmar.

## 2 Methods

### 2.1 Data source and sampling design

This study utilized secondary data from the Individual Recode (IR) file of the 2015–16 MDHS, the latest conducted by the Ministry of Health and Sports, Myanmar. The survey employed a stratified two-stage sampling approach. First, 442 clusters were chosen, 123 urban and 319 rural, from a pool of 4,000 primary sampling units (PSUs). Then, 30 households were selected from each cluster, totaling 13,260 households. Women aged 15–49 in these households were eligible for interviews, and in every second household, men aged 15–49 were also included. Data collectors were thoroughly trained by the DHS program experts, including interview techniques and procedures for completing the MDHS questionnaires (25).

Although the 2015–16 MDHS included 12,885 women aged 15–49 years, this study focused on 7,759 currently married women because questions used to assess attitudes toward negotiating safer sexual relations are most relevant within marital contexts, where Myanmar’s patriarchal norms and power dynamics are deeply embedded. This focus also ensures conceptual consistency and comparability with similar research (10, 13, 15, 23). After excluding 727 participants with missing values in all interested variables and 905 women who responded “do not know” to key outcome variables, the final sample size was reduced to 6,127.

### 2.2 Variables measurements

#### 2.2.1 Outcome variable

The outcome variable in this study was ‘Attitudes toward Negotiating SSR’ which was constructed from two separate questions: (1) Is a wife justified in refusing to have sex with her husband when she knows he has sex with other women? (2) If a wife knows her husband has a disease that she can get during sexual intercourse, is she justified in asking that they use a condom when they have sex? Women could answer ‘Yes,’ ‘No,’ or ‘Do not know,’ but those who chose ‘Do not know’ were excluded. Responses were coded as ‘negative attitude’ (0) if ‘No’ was given to either question or ‘positive attitude’ (1) if ‘Yes’ was given to both (10). This binary classification of attitudes was chosen due to the limited response options in the original dataset and for analytical clarity and consistency with prior studies (10, 13, 15, 23), despite this approach potentially oversimplifying women’s beliefs and experience.

#### 2.2.2 Explanatory variables

The variable selection was informed by the theoretical concepts from the Theory of Gender and Power (26), the Theory of Planned Behavior (17), and the findings from the prior studies. The independent variables were divided into three main categories: Socio-demographic factors included age (women and husband), child-marital status (getting married at the age of < or ≥ 18 years), fertility preference, wealth quintile, husband’s education level, region of residence (states/regions) classified accordingly to the 2008 constitutional framework of Myanmar (27); regions are primarily inhabited by the majority Burmese (also known as Bamar) population, while states are predominantly home to ethnic minority groups (28), place of residence (rural and urban), and household structure (polygamy/ monogamy) (29, 30).

Under the category of Health-related knowledge and myths, ‘knowledge of HIV’ transmission was measured using three variables: (1) reduce risk of getting HIV: always use condoms during sex, (2) have 1 sex partner only, who has no other partners, and (3) a healthy-looking person can have HIV. While a code ‘0 = Have no/partial knowledge’ was assigned if the respondent’s answer was ‘no or do not know’ to all questions, ‘1 = Have knowledge’ was given if her response was ‘yes’ to any of the three questions (18, 31). Another variable, ‘misconception about HIV transmission’, was also computed from three variables: (1) can get HIV from mosquito bites, (2) can get HIV by sharing food with a person who has AIDS, and (3) can get HIV by witchcraft or supernatural means. It was coded as ‘0 = Have misconception’ if the answer was ‘yes or do not know’ to any of the three questions; however, if the respondent answered ‘no’ to all questions, the code ‘1 = Have no misconception’ was assigned (18, 31). The final variable, ‘awareness of other STIs’, was derived from the question “Have you heard about other sexually transmitted infections?” Responses were coded as ‘0 = no or do not know’ and ‘1 = yes.’

Guided by the Theory of Gender and Power and the women’s empowerment framework, gender-power-mediating factors refer to variables reflecting how gender inequality and power relations shape women’s decision-making and agency in intimate relationships. Under this category, education, employment, and asset ownership represent structural power; decision-making autonomy, self-efficacy to refuse sex, and request condom use indicate interpersonal agency; while acceptance of wife-beating and household headship capture normative power shaped by societal and household norms (9, 18, 32). The ‘household decision-making power’ variable was computed from three original variables: (1) the person who usually decides on the respondent’s health care, (2) the person who usually decides on large household purchases, and (3) person who usually decides on visits to family or relatives. It was coded ‘0 = No’ if the respondent’s answer was ‘husband/partner alone or someone else or other’ in all variables. In contrast, it was coded as ‘1 = Yes’ if the respondent’s answer was ‘respondent alone or respondent and husband/partner or respondent and another person’ to any of the three variables (10, 18). ‘Self-efficacy to refuse sex’ was measured by the question, ‘Could you say no to your (husband/partner) if you do not want to have sexual intercourse?’, and ‘self-efficacy to request condom’ use was assessed by, ‘Could you ask your (husband/partner) to use a condom if you wanted him to?’. Another composite variable, ‘ownership of assets’ was constructed from two separate variables: (1) owns a house alone or jointly, and (2) owns land alone or jointly. The respondent’s answers could be in 4 categories: does not own/ /alone only/jointly only/both alone and jointly. If the respondent replied as ‘does not own’ to both questions, it was coded as ‘0 = Not owned’, if the response was otherwise to any of the questions, it was coded as ‘1 = Owned’ (18, 23).

In alignment with previous studies that have utilized the Demographic and Health Survey (DHS) data in comparable sociocultural contexts (10, 18, 31), we employed collapsed categories for some key variables to ensure methodological consistency, support comparability across studies, and ensure sufficient sample sizes for valid statistical analysis.

### 2.3 Statistical analysis

All analyses were conducted using SPSS version 26.0 (SPSS Inc., Chicago, IL, United States), and weighted samples were utilized in accordance with the DHS guidelines (33). Descriptive statistics were initially employed and reported in frequencies and percentages. Simple logistic regression was used to select variables for the final model based on the likelihood ratio and a *p*-value threshold of 0.1 from the Chi-square test. This step helped ensure that only potentially relevant variables were included and prevented overfitting in multiple logistic regression models.

The final step of model selection applied multiple regression analysis with the enter method. This was then validated by a stepwise method, with statistical significance determined by a *p*-value below 0.05, along with corresponding 95% confidence intervals. Three analytical steps were performed to examine factors associated with women’s attitudes toward negotiating SSR. Initially, each subgroup of independent variables was analyzed separately to identify significant predictors. Model 1 incorporated sociodemographic factors and health-related knowledge and myths, given evidence that greater awareness about HIV/STIs transmission and its risks enhances women’s agency in sexual decision-making. Model 2 further included gender-power-mediating factors to examine the changes in associations after controlling for gender-power dynamics. Model performance and goodness-of-fit were evaluated using the Akaike Information Criterion (AIC) and Pseudo R-square (McFadden) statistics. Despite Model 2, which included gender-power-mediating factors, having a higher AIC value than Model 1, both models were retained, as the aim was to examine how associations changed with the introduction of additional variables, rather than to identify the best-fitting model.

## 3 Results

### 3.1 Distribution of attitudes toward negotiating SSR among married women aged 15–49 years

Overall, 86.5% of the samples have positive attitudes toward negotiating SSR. Concerning sociodemographic factors, positive attitudes were most common among women aged 35 and above (49.8%) and the distribution of negative attitudes followed the same trend. Wealth status also influenced attitudes, with the richest quintile reporting the highest positive attitudes (22.8%) and the poorest showing the highest negative attitudes (24.8%). Women in urban areas, regions, monogamous households, and those whose husbands have a secondary education level demonstrated more positive attitudes.

Regarding health-related knowledge and myths, women with HIV transmission knowledge showed more positive attitudes (94.6%), while misconceptions were more prevalent in the negative group.

Gender-power-mediating factors revealed that positive attitudes were more observed among employed women (62.4%) compared to the unemployed group (37.6%). Women with higher education reported the second-highest positive attitudes (34.7%) and the lowest negative attitudes (5.1%). Additionally, positive attitudes were prevalent in women with the ability to refuse sex (86.9%) or request condom usage (76.8%). Household headship and asset ownership showed similar distributions of positive and negative attitudes. See Table 1.

**Table 1:** Descriptive analysis of attitudes toward negotiating SSR (*n* = 6,127).

Variable	Negative (*n* = 824, 13.5%) Frequency (%)		Positive (*n* = 5,303, 86.5%) Frequency (%)		*p* value
Sociodemographic factors					
Age					0.003
15–24	93	(11.3)	675	(12.7)	
25–34	267	(32.4)	1,984	(37.4)	
35 and above	464	(56.3)	2,643	(49.8)	
Husband’s age					0.703
Younger than wife	270	(32.8)	1,784	(33.6)	
Older than wife (1–5 years)	366	(44.4)	2,398	(45.2)	
Older than wife (6–10 years)	130	(15.8)	793	(15.0)	
Older than wife (>10 Years)	58	(7.0)	328	(6.2)	
Child marriage status					0.998
Yes	190	(23.1)	1,223	(23.1)	
No	634	(76.9)	4,080	(76.9)	
Fertility preference					0.026
No	531	(64.4)	3,297	(62.2)	
Yes	281	(34.1)	1,841	(34.7)	
Undecided	12	(1.5)	164	(3.1)	
Wealth quintile					<0.001
Poorest	204	(24.8)	880	(16.6)	
Poorer	160	(19.4)	1,022	(19.3)	
Middle	180	(21.8)	1,080	(20.4)	
Richer	166	(20.1)	1,110	(20.9)	
Richest	114	(13.8)	1,210	(22.8)	
Husband’s education level					<0.001
No education	102	(12.4)	491	(9.3)	
Primary	380	(46.1)	2,050	(38.7)	
Secondary	290	(35.2)	2,283	(43.1)	
Higher	46	(5.6)	416	(7.8)	
Do not know	7	(0.8)	63	(1.2)	
Region of residence					<0.001
State	229	(27.8)	1,115	(21.0)	
Region	595	(72.2)	4,188	(79.0)	
Place of residence					<0.001
Rural	654	(79.9)	3,657	(69.0)	
Urban	166	(20.1)	1,645	(31.0)	
Household structure					<0.001
Polygamy	66	(8.0)	243	(4.6)	
Monogamy	755	(91.6)	5,054	(95.3)	
Do not know	3	(0.4)	6	(0.1)	
Health-related knowledge and myths					
Knowledge of HIV transmission					<0.001
Have no/ partial knowledge	87	(10.6)	286	(5.4)	
Have knowledge	737	(89.4)	5,017	(94.6)	
Misconception about HIV transmission					<0.001
Have misconception	603	(73.2)	3,347	(63.1)	
Have no misconception	221	(26.8)	1,956	(36.9)	
Awareness of other STIs					N/A
No	0	0	0	0	
Yes	824	(100.0)	5,303	(100.0)	
Gender-power-mediating factors					
Working status					0.019
Unemployed	275	(33.4)	1,994	(37.6)	
Employed	549	(66.6)	3,309	(62.4)	
Education level					<0.001
No education	96	(11.6)	447	(8.4)	
Primary	461	(55.9)	2,455	(46.3)	
Secondary	226	(27.4)	1,840	(34.3)	
Higher	42	(5.1)	560	(34.7)	
Household decision-making power					0.163
No	45	(5.5)	232	(4.4)	
Yes	779	(94.5)	5,070	(95.6)	
Autonomy of spending on husband’s earning					0.031
Make no decision	102	(12.4)	586	(11.1)	
Make decision alone	320	(38.8)	1,835	(34.6)	
Make decision jointly	399	(48.4)	2,859	(53.9)	
Husband/partner has no earnings	3	(0.4)	23	(0.4)	
Self-efficacy to refuse sex					<0.001
No	167	(20.3)	614	(11.6)	
Yes	634	(76.9)	4,608	(86.9)	
Uncertain	23	(2.8)	81	(1.5)	
Self-efficacy to request condom usage					<0.001
No	223	(27.1)	576	(10.9)	
Yes	472	(57.3)	4,070	(76.8)	
Uncertain	129	(15.7)	656	(12.4)	
Acceptance of wife beating					0.335
Accept	92	(11.2)	554	(10.4)	
Not Accept	700	(85.0)	4,484	(84.6)	
Undecided	32	(3.9)	265	(4.8)	
Head of household					0.022
Male	735	(89.1)	4,569	(86.2)	
Female	90	(10.9)	733	(13.8)	
Ownership of assets					0.001
Not owned	227	(27.5)	1,767	(33.3)	
Owned	597	(72.5)	3,536	(66.7)	

### 3.2 Bivariate and multivariate associations of Myanmar women’s attitudes toward negotiating SSR with explanatory variables using crude odds ratios (ORs) and adjusted odds ratios (aORs)

Regarding sociodemographic factors, women aged 35 and above were less likely to report positive attitudes than those aged 15–24 in both crude (OR 0.783, 95% CI: 0.616–0.993) and adjusted models (aOR 0.763, 95% CI: 0.586–0.995). Women undecided about fertility had over twice the odds of positive attitudes compared to those without a preference. Wealth showed a strong gradient, with women in the richest quintile more likely to report positive attitudes than those in the poorest (OR 2.460, 95% CI: 1.926–3.143; aOR 1.886, 95% CI: 1.391–2.558). Women residing in regions and urban areas are also more likely to have positive attitudes than their counterparts in both analyses. Additionally, women in monogamous households had higher odds of positive attitudes than those in polygamous households.

Health-related knowledge and myths were also significant predictors. Women knowledgeable about HIV transmission were over twice as likely to report positive attitudes compared to those with no/partial knowledge, although the association slightly decreased after adjustment (OR 2.068, 95% CI: 1.607–2.661; aOR 1.864, 95% CI: 1.444–2.406). Similarly, the absence of misconceptions about HIV transmission was associated with higher odds of positive attitudes in both crude and adjusted models.

Gender-power-mediating factors significantly shaped attitudes. For instance, women with secondary education and higher education were more likely to report positive attitudes than those with no education, even after adjustment (aOR 1.439, 95% CI: 1.100–1.882 and aOR 2.043, 95% CI: 1.377–3.030, respectively). However, employed women were less likely to report positive attitudes compared to unemployed women. Self-efficacy was a key predictor, as women confident in refusing sex or requesting condom usage showed significantly higher odds of positive attitudes (e.g., requesting condom usage: OR 3.343, 95% CI: 2.788–4.008; aOR 2.769, 95% CI: 2.284–3.357). See Table 2.

**Table 2:** Simple and multiple logistic regression analysis of association between attitudes toward negotiating SSR and ***each sub-group of explanatory variables***.

Variable	Crude OR	(95% CI)	*p*-value	aOR	(95% CI)	*p*-value
Sociodemographic factors						
Age						
15–24	Ref			Ref		
25–34	1.019	(0.792–1.311)	0.884	1.031	(0.796–1.335)	0.818
35 and above	0.783	(0.616–0.993)	0.044	0.763	(0.586–0.995)	0.045
Fertility preference						
No	Ref			Ref		
Yes	1.057	(0.905–1.234)	0.487	0.925	(0.776–1.103)	0.387
Undecided	2.183	(1.209–3.940)	0.010	2.020	(1.106–3.691)	0.022
Wealth quintile						
Poorest	Ref			Ref		
Poorer	1.485	(1.185–1.862)	0.001	1.414	(1.125–1.777)	0.003
Middle	1.396	(1.121–1.738)	0.003	1.323	(1.056–1.657)	0.015
Richer	1.552	(1.241–1.939)	<0.001	1.349	(1.064–1.712)	0.014
Richest	2.460	(1.926–3.143)	<0.001	1.886	(1.391–2.558)	<0.001
Husband’s education level						
No education	Ref			Ref		
Primary	1.122	(0.883–1.426)	0.348	0.986	(0.772–1.259)	0.908
Secondary	1.637	(1.280–2.094)	<0.001	1.194	(0.922–1.547)	0.179
Higher	1.864	(1.287–2.702)	0.001	1.025	(0.682–1.542)	0.904
Do not know	2.007	(0.872–4.621)	0.102	1.918	(0.825–4.461)	0.131
Region of residence						
State	Ref			Ref		
Region	1.449	(1.227–1.711)	<0.001	1.451	(1.225–1.719)	<0.001
Place of residence						
Rural	Ref			Ref		
Urban	1.777	(1.485–2.127)	<0.001	1.397	(1.123–1.739)	0.003
Household structure						
Polygamy	Ref			Ref		
Monogamy	1.817	(1.370–2.411)	<0.001	1.579	(1.182–2.108)	0.002
Do not know	0.581	(0.139–2.424)	0.456	0.552	(0.129–2.362)	0.423
Health-related knowledge and myths						
Knowledge of HIV transmission						
Have no/partial knowledge	Ref			Ref		
Have knowledge	2.068	(1.607–2.661)	<0.001	1.864	(1.444–2.406)	<0.001
Misconception about HIV transmission						
Have misconception	Ref			Ref		
Have no misconception	1.592	(1.351–1.875)	<0.001	1.513	(1.282–1.786)	<0.001
Gender-power-mediating factors						
Working status						
Unemployed	Ref			Ref		
Employed	0.833	(0.714–0.973)	0.021	0.844	(0.720–0.990)	0.037
Education level						
No education	Ref			Ref		
Primary	1.143	(0.898–1.456)	0.227	1.071	(0.836–1.372)	0.587
Secondary	1.748	(1.348–2.267)	<0.001	1.439	(1.100–1.882)	0.008
Higher	2.886	(1.965–4.239)	<0.001	2.043	(1.377–3.030)	<0.001
Autonomy of spending on husband’s earning						
Make no decision	Ref			-	-	-
Make decision alone	1.004	(0.789–1.278)	0.973	-	-	-
Make decision jointly	1.254	(0.992–1.586)	0.058	-	-	-
Husband/partner has no earnings	1.154	(0.364–3.663)	0.808	-	-	-
Self-efficacy to refuse sex						
No	Ref			Ref		
Yes	1.982	(1.639–2.397)	<0.001	1.466	(1.195–1.797)	<0.001
Uncertain	0.954	(0.583–1.560)	0.851	0.934	(0.561–1.556)	0.794
Self-efficacy to request condom usage						
No	Ref			Ref		
Yes	3.343	(2.788–4.008)	<0.001	2.769	(2.284–3.357)	<0.001
Uncertain	1.965	(1.540–2.508)	<0.001	1.908	(1.485–2.452)	<0.001
Head of household						
Male	Ref			Ref		
Female	1.317	(1.043–1.662)	0.020	1.187	(0.934–1.510)	0.161
Ownership of assets						
Not owned	Ref			Ref		
Owned	0.760	(0.646–0.895)	0.001	0.869	(0.732–1.032)	0.109

### 3.3 Multilevel logistic regression models predicting Myanmar women’s attitudes toward negotiating SSR

Model 1 included sociodemographic factors and health-related knowledge and myths, while Model 2 further included gender-power-mediating factors to examine the changes in associations after controlling for gender-power dynamics. Although Model 2 represented the most comprehensive model with all key predictors, its relatively low Pseudo R^2^ value (0.062) indicates limited explanatory capacity, suggesting that additional unmeasured factors, such as cultural and behavioral, may also play a role in shaping women’s attitudes toward negotiating SSR. See Table 3.

**Table 3:** Multiple logistic regression analysis of association between attitudes toward negotiating SSR and explanatory variables.

Variable	Model 1			Model 2		
	aOR	(95% CI)	*p*-value	aOR	(95% CI)	*p*-value
Sociodemographic factors						
Age						
15–24	Ref			Ref		
25–34	0.989	(0.763–1.282)	0.935	0.996	(0.762–1.302)	0.977
35 and above	0.735	(0.564–0.959)	0.023	0.777	(0.585–1.030)	0.080
Fertility preference						
No	Ref			Ref		
Yes	0.920	(0.771–1.097)	0.353	0.903	(0.753–1.082)	0.268
Undecided	2.002	(1.094–3.665)	0.024	1.893	(1.028–3.488)	0.041
Wealth quintile						
Poorest	Ref			Ref		
Poorer	1.424	(1.132–1.791)	0.003	1.381	(1.092–1.747)	0.007
Middle	1.294	(1.032–1.622)	0.025	1.216	(0.963–1.535)	0.100
Richer	1.283	(1.010–1.631)	0.041	1.190	(0.928–1.526)	0.171
Richest	1.723	(1.267–2.343)	0.001	1.537	(1.117–2.114)	0.008
Husband’s education level						
No education	Ref			Ref		
Primary	0.930	(0.726–1.190)	0.562	0.914	(0.706–1.183)	0.495
Secondary	1.095	(0.842–1.423)	0.499	1.040	(0.789–1.371)	0.781
Higher	0.868	(0.573–1.315)	0.504	0.640	(0.405–1.011)	0.056
Do not know	1.805	(0.774–4.209)	0.171	1.899	(0.805–4.479)	0.143
Region of residence						
State	Ref			Ref		
Region	1.435	(1.209–1.702)	<0.001	1.442	(1.208–1.720)	<0.001
Place of residence						
Rural	Ref			Ref		
Urban	1.364	(1.096–1.698)	0.005	1.207	(0.964–1.511)	0.100
Household structure						
Polygamy	Ref			Ref		
Monogamy	1.554	(1.162–2.077)	0.003	1.524	(1.132–2.051)	0.005
Do not know	0.544	(0.125–2.360)	0.416	0.524	(0.119–2.308)	0.393
Health-related knowledge and myths						
Knowledge of HIV transmission						
Have no/partial knowledge	Ref			Ref		
Have knowledge	1.688	(1.300–2.193)	<0.001	1.495	(1.141–1.959)	0.004
Misconception of HIV transmission						
Have misconception	Ref			Ref		
Have no misconception	1.315	(1.100–1.573)	0.003	1.161	(0.964–1.398)	0.115
Gender-power-mediating factors						
Working status						
Unemployed	-	-	-	Ref		
Employed	-	-	-	0.849	(0.722–0.999)	0.049
Education level						
No education	-	-	-	Ref		
Primary	-	-	-	0.906	(0.697–1.178)	0.461
Secondary	-	-	-	1.038	(0.765–1.408)	0.810
Higher	-	-	-	1.427	(0.879–2.318)	0.151
Self-efficacy to refuse sex						
No	-	-	-	Ref		
Yes	-	-	-	1.388	(1.128–1.708)	0.002
Uncertain	-	-	-	1.042	(0.619–1.753)	0.878
Self-efficacy to request condom usage						
No	-	-	-	Ref		
Yes	-	-	-	2.713	(2.228–3.303)	<0.001
Uncertain	-	-	-	1.931	(1.496–2.492)	<0.001
Head of household						
Male	-	-	-	Ref		
Female	-	-	-	1.146	(0.898–1.463)	0.273
Ownership of assets						
Not owned	-	-	-	Ref		
Owned	-	-	-	0.926	(0.772–1.112)	0.412
Akaike’s information criterion	1931.694			4296.531		
Pseudo R-square (McFadden)	0.32			0.062		

#### 3.3.1 Sociodemographic factors

Women aged 35 and above had lower odds of reporting positive attitudes compared to the reference group in Model 1 (aOR 0.735, 95% CI: 0.564–0.959), but this association became nonsignificant in Model 2. Fertility preference consistently showed a significant association, with women undecided about fertility having higher odds of positive attitudes than those with no preference in both models. In both models, women in the poorer and richest wealth quintiles had significantly higher odds of a positive attitude than those in the poorest quintile, which remained significant in Model 2 (aOR 1.381, 95% CI: 1.092–1.747 and aOR 1.537, 95% CI: 1.117–2.114, respectively). Regional residence was also consistently associated with positive attitudes in both models. However, the significance of urban residence observed in Model 1 (aOR 1.364, 95% CI: 1.096–1.698) became nonsignificant in Model 2. Women in monogamous households were consistently more likely to report positive attitudes than those in polygamous households.

#### 3.3.2 Health-related knowledge and myths

Knowledge of HIV transmission was strongly associated with positive attitudes in both models, although the effect size decreased slightly after including gender-power-mediating factors (Model 1: aOR 1.688, 95% CI: 1.300–2.193; Model 2: aOR 1.495, 95% CI: 1.141–1.959). However, the absence of misconceptions about HIV transmission was significant in Model 1, but became nonsignificant in Model 2.

#### 3.3.3 Gender-power-mediating factors

Employment status was inversely associated with positive attitudes, as employed women were less likely to report positive attitudes than unemployed women (aOR 0.849, 95% CI: 0.722–0.999). Self-efficacy consistently emerged as a key determinant, with women confident in refusing sex showing significantly higher odds of positive attitudes (aOR 1.388, 95% CI: 1.128–1.708). The strongest association was observed for self-efficacy to request condom usage (aOR 2.713, 95% CI: 2.228–3.303), even among women uncertain about requesting condom usage. However, neither household headship nor asset ownership showed significant associations in Model 2.

## 4 Discussion

The findings from this study offer a comprehensive understanding of how multifaceted factors are associated with Myanmar women’s attitudes toward negotiating SSR. The foundational role of sociodemographic factors in shaping Myanmar women’s attitudes toward negotiating SSR was evident in both models. For instance, women in the richest quintile showed significantly higher odds of positive attitudes, consistent with the findings of previous studies (13, 23) while other studies showed contradicting findings (10, 15). This could be attributed to a higher economic status linked with greater access to health information and higher autonomy in healthcare decisions (34), underscoring the significance of economic empowerment in shaping women’s attitudes toward negotiating their sexual health. Interestingly, the odds of reporting positive attitudes were also high among women in the poorer quintile group, aligning with previous studies (10, 23). One plausible explanation may be the impact of potential exposure to targeted sexual and reproductive health interventions implemented by government and non-governmental organizations, particularly in urban slum settings where outreach efforts often concentrate on vulnerable populations.

Similarly, the likelihood of having positive attitudes among regional residents remained significant in both models compared to those residing in states. In Myanmar, ethnic minority groups predominantly live in seven ethnic states, which make up at least one-third of the total population (28). This may explain the structural disparities in access to resources and support systems between states and regions in Myanmar, which result in inequalities in accessing healthcare services (35), which can influence women’s attitudes. Another study that applied the Theory of Gender and Power in HIV preventive measures also mentioned that ethnic minority women are frequently expected to adhere to traditional gender roles, cultural expectations, and conservative religious values, which can shape their beliefs and potentially restrict their choices regarding sexual and reproductive health rights (26). These findings indicate the necessity of tailored and culturally sensitive initiatives for ethnic minority groups. The significance of positive association was high among urban residents in the initial model, which parallels findings from other studies (15, 18, 26, 31). However, it diminished in the final model, highlighting the need to recognize and address all the factors associated with women’s attitudes and their negotiation capacity for SSR.

This study also found that women in monogamous households consistently demonstrated higher odds of positive attitudes, aligning with the existing literature (2, 13, 29). This may be attributable to married women in polygamous families struggling to negotiate safer sex due to competition for their husbands’ affection and resources, fearing it may affect their love and respect (2). This suggests that patriarchal dynamics in polygamous households may limit women’s sexual autonomy and the importance of including men in addressing women’s sexual and reproductive health issues.

Health knowledge emerged as a strong predictor of possessing positive attitudes about safer sex negotiation. For instance, women knowledgeable about HIV transmission were more likely to report positive attitudes, even after adjusting for gender-power-mediating factors, aligning with other studies (18, 23). This could be attributed to the fact that women with sufficient HIV knowledge are more health-conscious, and their awareness of HIV risks reinforces the belief that negotiating safer sex is justified (23). However, the absence of misconceptions about HIV transmission resulted in higher odds of positive attitudes in Model 1, supporting a previous finding (31), however, this became nonsignificant in Model 2. This finding aligns with evidence that in patriarchal contexts, women’s STI awareness does not always translate into safer-sex negotiation due to religious, cultural, and power-related barriers (15). This shift suggests that while addressing misconceptions is important, considering gender-power dynamics is crucial to encourage women to negotiate safer sexual practices.

It was also found that self-efficacy was a particularly strong determinant, with women confident in refusing sex or requesting condom usage showing significantly higher odds of positive attitudes, aligning with a theoretical foundation explained in the Theory of Planned Behavior (17). This finding could explain the idea that human attitudes can influence their intention, choice, and behavior (16, 36, 37), and is also consistent with existing studies (38). Interestingly, women’s employment status showed an inverse association, similar to another Nigerian study (13), while other existing studies had found mixed results: a positive association (18), and no association (15, 18, 38). The possible explanation for this association may be the negative impact of women’s workplace exposure and the exchange of ideas regarding sexual and reproductive health (13), and may reflect the dual burden of employment and domestic responsibilities or power imbalances in relationships that working women face (39). This inverse relationship may also be due to male backlash in environments where traditional norms dominate, as employed women with more autonomy often face relational tension, reduced bargaining power at home, or stigma for stepping outside expected gender roles, and even increase vulnerability to violence (40) which may affect their sexual negotiation agency. Another study also explained that engaging in economic activities does not automatically lead to greater bargaining and negotiating power for women, as this can be influenced by the types of jobs in which women are involved (41). This highlights the importance of disaggregating employment types in future research to better understand how different forms of work influence women’s autonomy, capacity, and willingness to engage in safer sex negotiation. The finding highlights the need for targeted sexual risk prevention and reduction initiatives for working women. Beyond offering fair employment, it is also equally important to address gender power imbalances in the workplace by actively challenging discrimination and linking women to essential support services, including sexual health care, counseling, and gender-based violence resources.

After adjustment, education levels, household leadership, and asset ownership showed no significant association with positive attitudes; however, bivariate analysis indicated that women with higher education were more likely to believe the justification of negotiating SSR, similar to existing studies (10, 13, 18, 23, 31). This may be because higher education levels can increase health awareness and empower women to advocate for safer sex with their husbands (13). Nevertheless, the association between women’s education level and higher odds of positive attitude diminished in Model 2, proving that even educated women may have limited confidence to negotiate safer practices when considering gender-power-mediating factors. This observation may reflect Myanmar’s cultural norms, where discussing sex makes them feel embarrassed both at home and in public, and formal sex education is not taught at school (21). Another mixed-method study conducted in Myanmar found that while most students favored comprehensive sex education, parents and teachers typically supported an abstinence-only approach. Furthermore, overall reproductive health knowledge was low among all involved groups (42), providing a strong rationale for implementing school-based sexual health programs.

The key strength of this study is its use of a nationally representative dataset, ensuring broader applicability to all of Myanmar’s married women. A multilevel analysis approach further helps a comprehensive understanding of the complex factors associated with attitudes toward SSR. This study also has some limitations, including the cross-sectional nature of the survey, which can restrict causal relationships and temporal sequence, and self-report biases that can lead to social desirability bias in responses, particularly in sensitive topics like sexual negotiation. Some key behavioral and cultural determinants, like religious beliefs, exposure to gender-based violence, and relational dynamics, were also not able to be included in this study due to data availability limitations. Additionally, the relatively low Pseudo R^2^ value of Model 2 indicates limited explanatory power, suggesting that these unmeasured factors may also influence women’s attitudes toward negotiating SSR. Also, while the 2015–16 MDHS remains the most recent nationally representative data available, it may not fully reflect the current realities facing Myanmar women due to recent social, political, and economic transformations over the years.

Future studies should also consider including more comprehensive factors not covered in this study, and conducting in-depth qualitative and/or longitudinal research. Moreover, a notable portion of exclusions based on missing or non-informative data may have introduced selection bias and affected the generalizability of the findings in this study. Future research should consider conducting sensitivity analyses to examine the stability of these results under alternative data handling approaches. Additionally, although interaction effects were not tested in this study, future research is recommended to examine potential interactions to gain deeper insights into the combined influence of key factors on women’s attitudes toward negotiating SSR.

## 5 Conclusion

This study found that the majority of married women in Myanmar had positive attitudes toward negotiating safer sexual relations. These attitudes were strongly associated with factors such as women’s wealth status, their regional residence, household composition, knowledge about HIV transmission, and self-efficacy. Additionally, gender-power-mediating factors had a significant influence on the associations of sociodemographic and knowledge-related variables with the outcome variable. This finding is also consistent with existing literature showing that Myanmar women often face challenges in negotiating condom use with their partners, even when suspecting infidelity, and a woman’s awareness of STIs and their consequences may not lead to effective negotiation for safer sex in a patriarchal society. The fact highlights the need for gender-sensitive public health interventions that enhance women’s health knowledge, confidence, and negotiation skills in sexual health decision-making and promote economic empowerment. Addressing these factors within existing reproductive health programs can contribute to reducing HIV/STI transmission and improving sexual and reproductive health outcomes among women in Myanmar.

## Data Availability

Publicly available datasets were analyzed in this study. The datasets analyzed in this study are publicly available on the DHS program website upon reasonable request (https://dhsprogram.com/Countries/Country-Main.cfm?ctry_id=64&c=Myanmar&Country=Myanmar&cn=&r=4).
